# Renal Thrombotique microangiopathy: An unusual renal involvement in Niemann‐Pick disease type B

**DOI:** 10.1002/ccr3.3408

**Published:** 2020-10-19

**Authors:** Mouna Jerbi, Mariem Sayhi, Hanene Gaied, Hafedh Hedri, Raja Aoudia, Rim Goucha, Taieb Ben Abdallah

**Affiliations:** ^1^ Nephrology Department CHU Mongi Slim La Marsa Tunisia; ^2^ Faculty of Medicine University of Tunis El Manar Tunis Tunisia; ^3^ Medicine A Department Charles Nicolle Hospital Tunis Tunisia

**Keywords:** endocrinology and metabolic disorders, hematology, nephrology

## Abstract

Renal involvement in Niemann‐Pick disease type B is very rare. Kidney check‐up and renal biopsy should be performed in any patient presented with hypertension and kidney disease. Histology identifies the lesion, the prognosis, and guide treatment.

## Graphical abstract

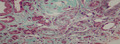

## 1 BACKGROUND

Niemann‐Pick disease (NPD) is an autosomal recessive disorder. It is characterized by intracellular deposition of sphingomyelin.

We mention the most common forms of the disease: NPD type A is the neuropathic type. It occurs from birth in an acute way and progresses rapidly to death. NPD type B is the form which has the best prognosis and the best survival in adulthood. This form does not present neurological damage. NPD type C appears at a very variable age. This form can affect several organs (liver, spleen, and lung). It also affects the nervous system with sometimes severe impairment.^1^

NPD type B is the less severe form of the disease. Its etiology is mutation in the sphingomyelin phosphodiesterase‐1 gene (SMPD1) which causes a primary deficiency of acid sphingomyelinase (ASM) activity. It is characterized by phenotypic heterogeneity.^2^ Accumulation of sphingomyelin can affect many tissues and leads to severe morbidity especially due to pulmonary, cardiac, and hepatic impairment.^3^ Renal involvement of NPD has been rarely described.^4^

We present a case of NPD type B with renal Thrombotic microangiopathy (TMA) was never described in literature before. Is it a fortuitous association or rather a new manifestation of the disease?

## 2 CASE REPORT

The patient was a 29‐year‐old Caucasian man with no family history. He has a past medical history of unexplained cervical lymphadenopathy and splenomegaly. He was hospitalized to investigate a chronic kidney failure. The initial workup revealed high blood pressure (170/100 mmHg) and splenomegaly; no neurological or ophthalmic findings were noted. He had no growth retardation. Low proteinuria (1+) was detected on urinalysis without hematuria. Laboratory studies revealed the following: serum creatinine 1000 µmol/L, hemoglobin 8 g/dL, platelet count was normal, hypertriglyceridemia 5 mmol/L, and low HDL cholesterol 0.35 mmol/L. Liver function was normal. Anemia was not hemolytic with absence of schizocytes and normal lactate dehydrogenase (LDH) and bilirubin levels. Viral serologies were negative (cytomegalovirus, Epstein‐Barr virus, hepatitis C and B, HIV, and parvovirus). Antinuclear antibodies were also negative, and serum protein electrophoresis was normal.

Abdominal ultrasound revealed splenomegaly (22 centimeters) with some hypodense nodules, two small kidneys with bad corticomedullary differentiation. Transthoracic echocardiography did not reveal signs of left ventricular hypertrophy. Left ventricular systolic function was low (ejection fraction = 45%). Computed tomography of the lungs did not show any interstitial pattern.

**Figure 1:**
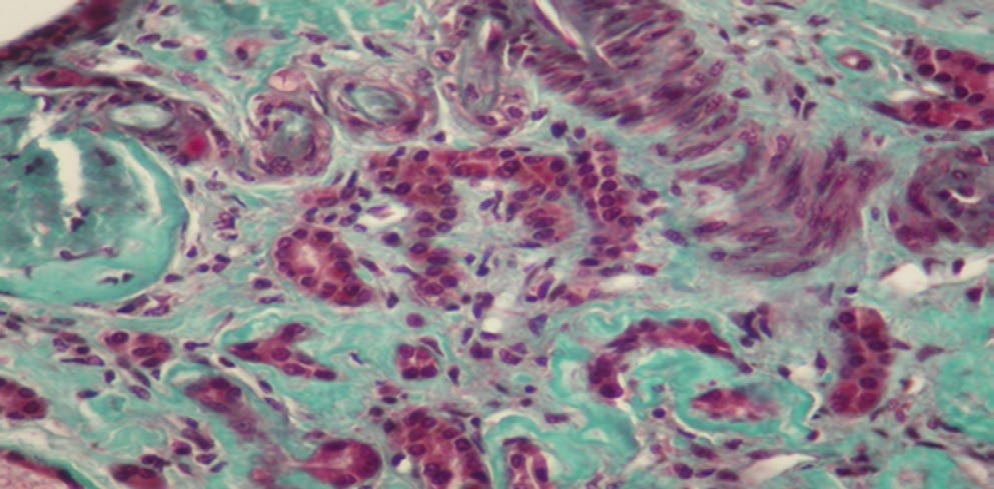
Renal biopsy. Masson’s trichrome (magnification × 200): 2 thrombotic microangiopathy lesions

**Figure 2:**
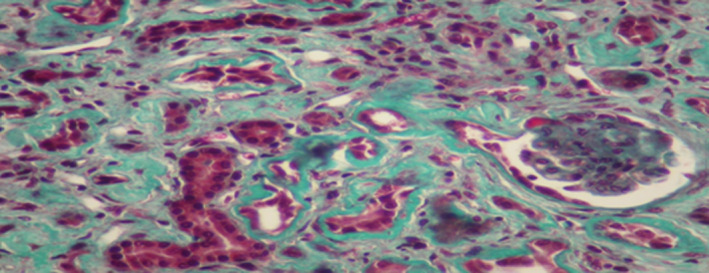
Renal biopsy. Masson’s trichroma: collapsing focal segmental sclerosis lesion

**Figure 3:**
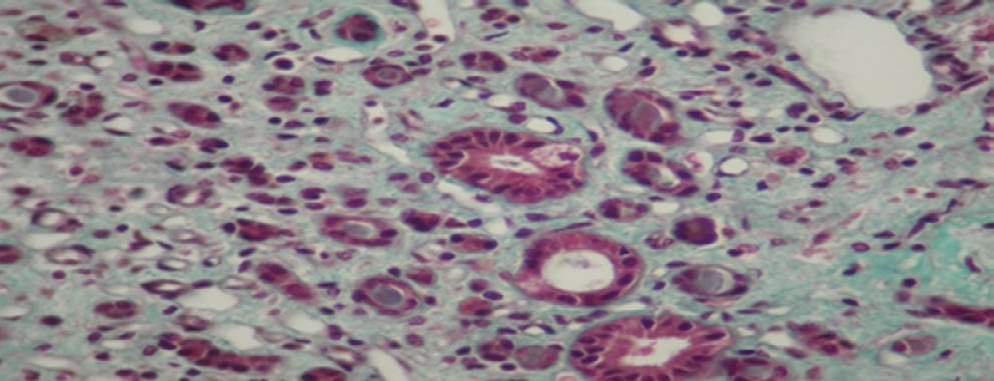
Renal biopsy. Masson’s trichroma: foamy cell in tubular epithelium

**Figure 4:**
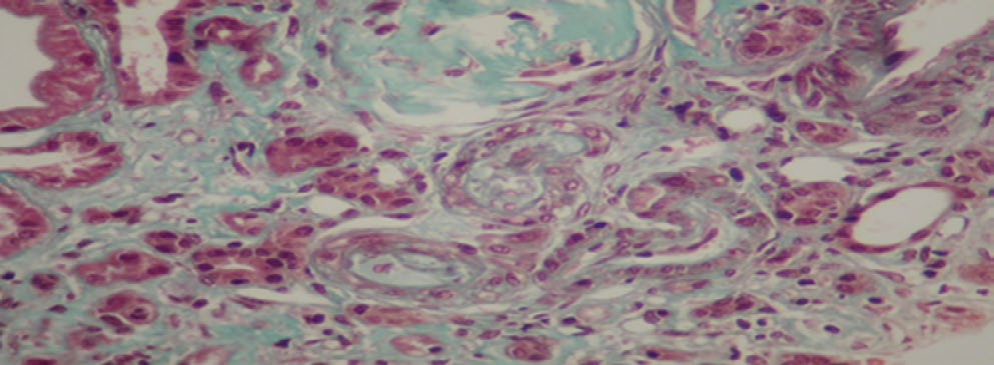
Renal biopsy. Masson’s trichroma: TMA with foamy cell in vessel

The patient had renal biopsy (Figures 1,2,3,4). The renal biopsy specimen was examined by light microscopy using conventional staining methods and immunofluoresence. The obtained renal tissue contained 28 glomeruli: 24 were sclerotic and atrophic. Only 4 glomeruli were permeable. There was no marked mesangial cell proliferation. The glomeruli showed mesangial changes with variable degree of mesangial matrix expansion. Three glomeruli contained focal segmental sclerosis (FSGS) with enlarged and vacuolated podocytes and some podocytes foot process. One glomerulus contained a collapsing focal segmental glomerulosclerosis. There were some foamy podocytes in glomeruli. Many sclerotic glomeruli presented thrombotic microangiopathy lesions. The interstitial tissue showed expanded fibrosis, tubular atrophy with disappearance of the brush border, and extensive tubular casts. Moreover what was more striking in the biopsy was severe vascular lesions. There were severe thrombotic microangiopathy (TMA) lesions. The arteries and arterioles appeared with endothelial dysfunction and thickening of their wall and tiny lumen. These arterioles had an "onion skin" appearance. In addition, we noted some foam cells in tubular epithelium and in arterial vessels and many vacuolated tubular epithelial cells. Immunofluorescence revealed no deposition along the capillary wall but some C3 deposits in 2 glomeruli.

In conclusion, the advanced lesions present during the renal biopsy made it difficult to classify the nephropathy. There were some FSGS type collapsing with enlarged podocytes and some foamy podocytes, severe interstitial damage, foam cells in tubular epithelium, and in arterial vessels, many vacuolated tubular epithelial cells and especially severe vascular lesions as TMA lesions.

We performed later bone marrow aspiration for a thrombocytopenia which appeared secondarily. It revealed sea blue histiocytosis.

NPD type B was suspected, it was confirmed when residual activity of ASM in peripheral blood leukocytes was measured (ASM activity = 0.16 UI [normal range = 042‐0.92]). The blood sample for molecular study has been realized but we have no result yet because of technical problems.

The patient was on end‐stage renal disease (ESRD). The patient was treated by conventional hemodialysis. After 2 years of follow‐up, he is doing well.

Some typical clinical features of NPD type B were present: splenomegaly with thrombocytopenia, lipid abnormalities, and no neurological abnormalities. What was atypical is the lack of interstitial lung disease. Our patient had proofs of renal involvement of NPD which were the foamy podocytes in glomeruli and foam cells in tubular epithelium and in arterial vessels, but had also another significant and not common histologic lesion as renal TMA never reported in literature before.

## 3 DISCUSSION

NPD type B is a rare disease. Its prevalence is estimated to be 1/230 000 in France.^5^ The diagnosis is often made in childhood. In the Lidove and al Series’,^5^ the diagnosis is made before age 30 in 57 % of cases, at a median age of 11.5 years. It can appear at any age from childhood to the 5th decade.^3^ In our case, the disease was progressing slowly, probably since childhood, but age at diagnosis was 29 years old.

It is a multisystemic disease. The most common manifestations of the disease are hepatosplenomegaly, hyperlipidemia, and infiltrative pulmonary lesion. Other lesions can be described: liver dysfunction, cardiac disease, retinal stigmata, growth retardation, and skeletal manifestations including osteoporosis and osteopenia. Neurological involvement may be present with varying manifestations including ataxia, developmental delay, and peripheral neuropathy.^3^ In a series of 103 patient reported by Mc Govern and coll, 13 patients had neurological impairment.^3^

Also, lipid abnormalities are frequently described in NPD type B. It is about hypertriglyceridemia, decreased high‐density lipoprotein (HDL) cholesterol, and increased low‐density lipoprotein (LDL) cholesterol. The mechanism of this dyslipoproteinemia is not entirely understood. These abnormalities can be associated with atherosclerotic vascular disease and coronary artery disease.^6^

Serious morbidities included clinically significant hepatic disease can be present in NPD type B which can lead to end‐stage liver failure and the need for a liver transplantation. We add also some cases of cirrhosis. He was evoked that pathologic intralysosomal storage of sphingomyelin may cause a fibrotic reaction in tissues, leading to liver dysfunction.

Another serious morbidity is interstitial lung disease. It is about recurrent respiratory infections^7^ Pulmonary involvement can progress to pulmonary failure with oxygen dependance. The pulmonary disease is explained by the accumulation of sphingomyelin in the alveolar macrophages. However, studies in ASM knockout mice have shown that abnormal surfactant composition and inflammation may additionally cause pulmonary disease.^8^ Lung biopsies realized in adults with NPD type B have shown endogenous lipid pneumonia, interstitial fibrosis, and accumulation of foamy macrophages.^3^

So NPD type B can be very dangerous and vital prognosis can be engaged. Markers of poor prognosis are liver failure, pulmonary infections with oxygen dependency, hemorrhage, splenic rupture, coronary artery, and valvular heart disease.^8^, ^9^

Our patient had splenomegaly without other organomegaly, anemia, thrombocytopenia, and lipid abnormalities with low left ventricular systolic function without ventricular hypertrophy or coronary disease. He had no growth retardation, no infiltrative pulmonary disease nor liver dysfunction, or retinal stigmata.

Considering these clinical features, we eliminated first viral infections as cytomegalovirus, Epstein‐Barr virus, and parvovirus. Our patient also had hematological abnormalities and renal dysfunction, and it was necessary to eliminate systemic lupus erythematosus (SLE) and myeloma. In fact, antinuclear antibodies were negative and serum protein electrophoresis was normal.

Then, we performed marrow bone aspiration because of unexplained thrombocytopenia which revealed sea blue histiocytosis. By combining signs and symptoms, we suspected a storage disease. We reviewed the literature and found that Lidove O and al described sea blue histiocytosis in 8 patients out of 28 patients with NPD type B.^5^ The diagnostic puzzle was resolved when our patient showed a reduced level of sphingomyelinase activity as seen in type B form. Unfortunately, we have no genetic studies. In fact, NPD type B is an allelic disorder caused by mutation in the sphingomyelin phosphodiesterase‐1 gene (SMPD1). The diagnosis of ASM deficiency can also be confirmed by molecular genetic study. It identifies disease‐causing alleles. This study is usually performed after biochemical testing for ASM activity. Gaucher disease was also evocated but glucocerebrosidase activity was not assessed since the appearance of sea blue histiocytosis was strongly evocative of NPD.

Otherwise, our patient had another significant clinical event exceptionally reported in the literature in patients with NPD type B. In fact, our patient had a chronic renal disease on end‐stage renal failure. It probably was a vascular nephropathy. He had hypertension and low proteinuria. Renal biopsy showed advanced lesions with expanded interstitial fibrosis and sclerotic glomeruli, some FSGS type collapsing and especially severe TMA lesions. There were also some foamy podocytes, foam cells in tubular epithelium and in arterial vessels. The question was is this fortuitous association or rather a new manifestation of the disease?

In literature, renal involvement has only been described in some cases of NPD type A and NPD type B. Foam cells were found in glomerular on post‐mortem kidney biopsies.^10^ Renal involvement is more commonly described in other sphingolipidoses such as Fabry’s disease. It is characterized by accumulation of glycosphingolipid and cholesterol in glomerular cells and tubes.

After literature review, we found that renal involvement of NPD type B is rarely reported (2 cases of NPD type B). In fact, many cases of liver failure were reported but few cases of renal failure in NPD type B. Also few cases of kidney biopsy in NBD type B were reported. Furthermore, we found 3 other cases of renal involvement in NPD (2 cases of NPD type C and 1 case of NPD type E) (Table 1).

**Table 1:** Literature review if renal involvement in Niemann‐Pick disease (NPD)

Year	Author	Patient age	Gender	NPD	Renal parameters	Renal biopsy
1976	Briere J	26	Female	NPD type B	Proteinuria, hematuria, Moderate renal failure	Membranoproliferative glomerulonephritis type I and foamy cells in the glomeruli and tubular epithelium
2002	Jean Baptiste Philit	34	Male	NPD type C	Proteinuria, hematuria Hypertension, Moderate renal failure	Membranoproliferative glomerulonephritis type II with foamy cells in interstitial tissue
2009	Grafft CA	14	Female	NPD type B	Moderate Renal failure	Focal global glomerulosclerosis, tubular atrophy, interstitial fibrosis and vascular sclerosis, foamy podocytes, vacuolated tubular epithelial cells, and foamy cells in the interstitium
2016	Zhenda Zheng	43	Female	NPD type E	Proteinuria, hematuria, Moderate renal failure	Glomerulosclerosis with diffused foamy cells
2017	Mhamdi Alaoui	14	Female	NPD type C	Moderate Renal failure Tubular proteinuria	Tubular nephropathy with foamy cells (sea blue histiocytes) in epithelium tubular cells

In 1976, Briere was the first author who reported the case of a 26 years old woman whose first symptoms appeared when she was 17 months old. She had symptoms of hepatosplenomegaly and pulmonary infiltration. The diagnostic of NPD type B was confirmed by the presence of lipid laden macrophages (resembling foam cells, sea blue histiocytes) in the bone marrow and liver, an excess of tissue sphingomyelin and cholesterol, and a decrease in sphingomyelinase in circulating leukocytes. She developed membranoproliferative glomerulonephritis (MPGN) type I. The etiological investigation of this renal lesion found hypocomplementemia and chronic Staphylococcus aureus sepsis. However, MPGN regressed after antibiotic therapy. It is true that this glomerular nephropathy was not due to the storage disorder, although kidney biopsy showed in addition of MPGN, storage cells in the glomeruli, and tubular epithelium, with the accumulation of abnormal sphingomyelin and cholesterol.^11^

In 2002, Jean Baptiste Philit reported the case of a male born in 1965. At the age of 3, he developed hepatosplenomegaly. The diagnosis of NP disease had been suspected because of foam cells on the splenogram. At age of 31, he developed renal failure with signs of glomerular nephropathy (proteinuria = 4 g/24 h, microscopic hematuria). A renal biopsy showed MPGN type II with intense C3 staining along the capillary walls and double‐contour linear configuration. Light microscopy revealed mesangial proliferation, increased mesangial matrix, lobular accentuation, and capillary wall thickening. In the interstitium, there were some foam cells. Diagnostic confirmation required electron microscopy which had shown intramembranous electron‐dense deposits, without foam cells.^12^

In 2009, Grafft CA and coll reported the case of a girl with NPD type A/B. The disease was discovered at the age of 6 months. At the age of 14, she developed glomerular nephropathy. A kidney biopsy revealed focal global glomerulosclerosis with histological lesions of chronicity (vascular sclerosis, tubular atrophy, and interstitial fibrosis). Also, lesions related to the storage disease were seen: foamy podocytes and foamy cells in the interstitium. Electron microscopy revealed tubular epithelial cells, myelin‐like inclusions in podocytes, and endothelial cells.^4^

In 2016, Zhenda Zheng and coll published an article about a 43 years old Chinese female patient hospitalized with complaints of abdominal distension, weakness, anorexia, and weight loss of 5 kg in a month. The physical examination was normal. Laboratory findings showed that serum lipids were decreased especially high‐density lipoprotein. She had anemia with hemoglobin levels at 9.5 g/dl. She also had a glomerulopathy with proteinuria 2.5 g/24 h, and hematuria. Renal function was decreased (serum creatinine 3.7 mg/dL). Here is what the renal biopsy revealed on light microscopy: 8 slomerulosclerosis (8/12), widespread cytoplasmic vacuolation of endothelial and visceral epithelial cells in non‐sclerotic glomeruli, widespread vacuolar degeneration, focal dissolve and desquamation in proximal convoluted tubule, protein casts, and diffused foam cells in interstitial tissue. But, the chronic changes such as interstitial fibrosis or tubular atrophy were absent, and inflammatory cell infiltration was not obvious. The electronic microscopy showed massive lipidic deposits in endothelials and podocytes. Immunofluorescence showed the following: IgG(‐), IgA(‐), IgM(+), C3(‐), C1q(‐), and Fg(+). Liver biopsy showed swollen hepatocytes and widespread foam cells. The following signs (pathological changes of kidney and liver, the existence of NP cells in bone marrow, normal acid sphingomyelinase activity, and increased chitotriosidase activity) confirmed the diagnosis of NP disease of type E.^13^

In 2017, Mhamdi Alaoui reported in his doctoral dissertation the case of a 14 years old girl with NPD type C with renal impairment. She had tubular proteinuria with moderate renal failure. The obtained renal tissue contained one sclerotic glomerulus. The interstitial tissue showed fibrosis band with inflammatory cells, lympho‐plasmocyte cells, and many foamy cells. Most tubules were atrophic (> 50%), expanded and contained hematic casts. There were also foamy cells (sea blue histiocytes) in tubular epithelium cells and in arterial vessels.^14^

In all cases, we can notice that a renal biopsy showed glomerular and tubulo‐interstitial lesions with foamy cells.

We also notice that all patients had moderate renal failure and no one was in ESRD.

In our case, renal biopsy showed glomerular lesions as FSGS like in cases reported below. It also showed expanded interstitial fibrosis tubular atrophy similar to cases in the literature but TMA renal lesions have never been reported before. So the question is: Is it a fortuitous association or the first case of renal TMA associated with NPD type B?

In addition, we noted some foamy podocytes and foam cells in tubular epithelium and in arterial vessels in the biopsy of our patient which likely makes the link between the disease and the renal damage. Therefore, the disease has really affected the kidney since storage cells were present in the biopsy. Moreover, our patient’s biopsy was performed very late. In fact, he was in ESRD and had advanced histological lesions with many sclerotic glomeruli and expanded fibrosis which made the interpretation of lesions very difficult.

Otherwise, we did not find any biological TMA abnormalities. Infact, anemia was not hemolytic with absence of schizocytes and normal lactate dehydrogenase (LDH) and bilirubin levels. So TMA was only an histological finding. We also carried out an etiological investigation which was negative. In fact, our patient had not malignant hypertensions, auto‐immune disease, cancer nor history of radiotherapy… We used TMA classification of Buob and coll established in 2016, which included the following: hypertension, atypical hemolytic uremic syndrome, drugs, after renal transplantation, postpartum, antiphospholipid syndrome, drepanocytosis, postradiotherapy, and Castelman disease.^15^

So the question is: what is the link between TMA lesions and NPD?

We formulated two hypotheses, the first one was that the disease was the cause of this histological lesion. So, we suppose that since there were no causes of this histological lesion in our patient, NPD can be the cause. The following question arises: what is the pathophysiologic mechanism of TMA in NPD? We suppose that lipid abnormalities frequently described in NPD type B can be the explanation. These abnormalities can be associated with atherosclerotic vascular disease and coronary artery disease.^6^ They could cause thrombotic lesions in the kidney, especially since there was evidence of the impact of the disease on the kidney (foamy cells).

The second hypothesis is that there was no link between TMA lesions and NPD. Histological findings could be caused by vascular nephropathy probably under‐diagnosed for a long time. The patient probably had persistent high blood pressure for a long time. So it is a fortuitous association.

Finally, we conclude that renal involvement in NPD type B is not very common (2 cases). They were 2 cases of glomerular disease. But also, TMA lesions may be observed in NPD type B; which has never been reported in literature. It is associated with very poor renal prognosis since the patient was immediately at ESRD.

To date, no treatment is available, only symptomatic therapy. A specific treatment by the deficit enzyme is being evaluated.^16^ Our patient was treated with hemodialysis for his chronic renal insufficiency.

However, despite the rarity of renal involvement in NPD, it should be considered in all patients with this condition, especially in those with renal insufficiency. Monitoring of renal parameters in these patients as well as performing a kidney biopsy if abnormalities were to be found is recommended.

## 4 CONCLUSIONS

NPD type B is a rare storage disease. It is a multisystemic disease characterized by its clinical variability. Renal involvement of NPD type B is rarely reported, but it should be considered in all patients with this condition. It can be a glomerular disease and in our case a vascular disease (TMA). It aggravates the prognosis of the disease.

## CONFLICT OF INTEREST

None declared.

## AUTHOR CONTRIBUTIONS

All authors: involved in the conception of the work. Mouna Jerbi and Mariem Sayhi: wrote the first draft of the manuscript based on conversations with all authors. All authors: provided intellectual content, edited the manuscript, approved the final version for submission, and agree to be accountable for all aspects of the work.

## Data Availability

All patient data are available.
